# Transcription factor Krüppel‐like factor 4 upregulated G protein‐coupled receptor 30 alleviates intestinal inflammation and apoptosis, and protects intestinal integrity from intestinal ischemia–reperfusion injury

**DOI:** 10.1002/iid3.940

**Published:** 2023-07-27

**Authors:** Jie Yin, Xiaoli Xie, Jinfeng Yao, Xiaoxu Jin, Huiqing Jiang, Chenguang Ji

**Affiliations:** ^1^ Department of Gastroenterology The Second Hospital of Hebei Medical University Shijiazhuang Hebei China

**Keywords:** G protein‐coupled receptor 30, intestinal integrity, intestinal ischemia–reperfusion injury, Krüppel‐like factor 4, pyroptosis

## Abstract

**Introduction:**

Intestinal ischemia/reperfusion (I/R) injury is a common clinical event occurring during multiple clinical pathological processes. Here, we designed this paper to discuss the role of G protein‐coupled receptor 30 (GPR30) playing in intestinal I/R injury.

**Methods:**

An oxygen‐glucose deprivation/reoxygenation (OGD/R) cell model was established to simulate the pathological process of I/R injury. With the application of enzyme‐linked immunosorbent assay, TUNEL, and transepithelial electrical resistance (TEER) assays, the levels of inflammatory cytokines, cell apoptosis, and intestinal integrity were estimated. The corresponding proteins were estimated by applying western blot. Immunofluorescence was conducted to examine N‐terminal Gasdermin D (GSDMD‐N) expression. The interplay between KLF4 and GPR30 was demonstrated by dual‐luciferase reporter assay and chromatin immunoprecipitation.

**Results:**

The results showed that GPR30 was downregulated in Caco‐2 cells exposed to OGD/R. GPR30 overexpression reduced the production of TNF‐α, IL‐6, IL‐1β, and IL‐18, the TUNEL‐positive cells, as well as the contents of p‐p65, Cox‐2, Inos, Bax, and cleaved‐PARP, but elevated the expression of Bcl‐2 in OGD/R‐induced Caco‐2 cells. In addition, OGD/R‐induced the reduction of TEER value and reduced expression of tight junction proteins in Caco‐2 cells, which was partially restored by GPR30 overexpression. Furthermore, GPR30 suppressed nod‐like receptor pyrin 3 inflammasome and GSDMD‐N expression. It was evidenced that Krüppel‐like factor 4 (KLF4) could directly bind to GPR30 promoter and positively regulate GPR30 expression. The regulation of GPR30 overexpression above was weakened by KLF4 knockdown.

**Conclusion:**

Collectively, our findings suggested that KLF4 could transcriptionally upregulate GPR30, and GPR30 prevented intestine I/R injury by inhibiting inflammation and apoptosis, and maintaining intestinal integrity that provides potential targets for mitigating the I/R injury.

## Graphical abstract

- G protein‐coupled receptor 30 (GPR30) protects against oxygen‐glucose deprivation/reoxygenation‐induced inflammatory response, apoptosis, and intestinal integrity disruption.

- Transcription factor Krüppel‐like factor 4 (KLF4) directly binds to GPR30 promoter and positively regulates GPR30 overexpression.

- KLF4/GPR30 axis exerts function in intestinal ischemia/reperfusion injury partly by inhibiting the nod‐like receptor pyrin 3‐mediated pyroptosis.

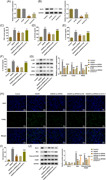

## 1 INTRODUCTION

Intestinal ischemia/reperfusion (I/R) injury is a common and severe clinical syndrome that occurs during large amounts of clinical emergencies such as intestinal obstruction, abdominal aortic aneurysm surgery, sepsis as well as intestinal transplantation.^1^, ^2^ Generally, Intestinal ischemia results in elevated microvascular permeability and destruction of the intestinal mucosal barrier, which promotes the translocation of intestinal bacteria or toxins, accompanied by a large number of inflammatory cytokines into the blood, causing not only local injury to the intestine itself, but also damage to distant organs, eventually contributing to systemic inflammatory reactions and multiorgan dysfunction. Restoration of blood supply via reperfusion therapy is a common treatment in clinical practice after intestinal ischemia; however, the secondary damage caused by reperfusion following ischemia leads to more severe functional impairments, which mainly accounts for the high morbidity and mortality associated with intestinal I/R injury.^3^, ^4^ Therefore, it is of great significance to explore the mechanisms of intestinal I/R injury and to find effective methods for preventing intestinal I/R injury.

G protein‐coupled receptor 30 (GPR30), which is called G protein‐coupled estrogen receptor 1 (GPER1) as well, is newly recognized as a membrane‐associated receptor.^5^ GPR30 is abundantly present in various tissues and cells.^6^ Activation of GPR30 has been reported to reduce the inflammatory response of macrophages, suggesting that GPR30 may be a therapeutic target for inflammatory diseases.^7^ Emerging evidence reveals that GPR30 is involved in regulating the pathological process of I/R injury of various types of tissue, such as liver tissues and myocardial tissues, and activation of GPR30 exerts a protective role in these I/R injuries.^8^, ^9^ Nevertheless, whether GPR30 also has involvement in intestinal I/R injury remains obscure. Of note, the expression level of GPR30 was observed to be abnormally low in patients with inflammatory bowel disease, and GPR30‐specific inhibitor G15 impaired the protective effects of dehydroepiandrosterone on the intestinal barrier and inflammatory response in mice with colitis, reflecting that GPR30 played a key role in intestinal injury.^10^, ^11^ Therefore, we speculate that GPR30 has involvement in intestinal I/R injury.

Krüppel‐like factors (KLFs) belong to the zinc finger protein family, which plays important roles in the transcriptional regulation of eukaryotic cells and acts as critical players in regulating various cellular processes, such as cell proliferation and differentiation.^12^ Transcription factor KLF4 is one of the most widely studied members of KLF family. It is worthy that KLF4 is also involved in regulating I/R injury in a variety of tissues. For instance, lncRNA MEG3 affects brain I/R injury by modulating macrophage polarization through binding to KLF4.^13^ Downregulation of miR‐34a ameliorates renal I/R‐induced inflammatory response and apoptosis by promoting KLF4 expression.^14^ In addition, sRC‐3 can reduce sepsis‐induced intestinal injury by enhancing the expression of the transcription factor KLF4, indicating a potential role of KLF4 in intestinal injury^15^; nevertheless, the role of KLF4 playing in intestinal I/R injury remains elusive.

Of note, using the Human TFDB website (http://bioinfo.life.hust.edu.cn/HumanTFDB/#!/), we found that transcription factor KLF4 was able to bind to the GPR30 promoter region, implying that KLF4 could potentially interact with GPR30. Therefore, we speculate that transcription factor KLF4 can potentially affect GPR30 expression levels by binding to the GPR30 promoter region, and thus participate in regulating the pathological process of intestinal I/R injury. Hence, this study aims to elucidate the role of GRP30 and its potential regulatory mechanism in intestinal I/R injury, which will provide a novel angle and target for intestinal I/R injury treatment.

## 2 MATERIALS AND METHODS

### 2.1 Cell culture

Caco‐2 cells provided by American Type Culture Collection (HTB‐37) were cultivated in Dulbecco's modified Eagle's medium supplemented with 20% fetal bovine serum and 1% penicillin‐streptomycin in a humidity incubator containing 5% CO_2_ at 37°C.

### 2.2 Oxygen‐glucose deprivation/reoxygenation model construction

An oxygen‐glucose deprivation/reoxygenation (OGD/R) cell model was established to simulate the pathological process of I/R as previously described.^16^ In brief, Caco‐2 cells were incubated with glucose‐free Earle's Balanced Salt Solution (EBSS; Leagene Biotechnology) in microaerophilic equipment with 1% O_2_, 5% CO_2_, and 94% N_2_ for 8 h and further incubated with glucose‐high EBSS in the conventional incubator (5% CO_2_, 95% air) for 20 h for reoxygenation.

### 2.3 Cell transfection

The GPR30 over‐expressing plasmid (oe‐GPR30), KLF4 over‐expressing plasmid (oe‐KLF4) as well as their corresponding negative control (oe‐NC) were provided by GenePharma. Short hairpin RNA (sh) targeting KLF4 (sh‐KLF4‐1 and sh‐KLF4‐2) and its negative control (sh‐NC) were also synthesized by GenePharma. 1 × 10^5^ Caco‐2 cells were inoculated into six‐well plates. When cells reached 70%–80% confluence, the transfection of above plasmids into cells was implemented by adopting Lipofectamine 3000 (Wuhan Kehaojia Biotechnology Co., Ltd.) in light of recommended protocols. Forty‐eight hours post‐transfection, cells were harvested for experiments.

### 2.4 PCR extraction and real‐time quantitative PCR

Total RNA was separated by applying TRIzol kit (Shanghai Absin Biotechnology Co., Ltd.) in light of standard specifications. The RNA purity as well as concentration was decided on adopting NanoDrop 3000 (Shanghai Aiyan Biotechnology Co., Ltd.). Then, 1 μg of total RNA was reverse‐transcribed into cDNA in light of the protocol of the reverse transcription kit (Takara), followed by the quantification of mRNA level using ChamQ Universal SYBR qPCR Master Mix (Vazyme Biotech Co., Ltd.) in light of the recommended specification. The sequences of the primers were as follows: GRP30, forward, 5′‐AGTCGGATGTGAGGTTCAG‐3′ and reverse, 5′‐TCTGTGTGAGGAGTGCAAG‐3′; KLF4, forward, 5′‐ACCTACACAAAGAGTTCCCATC‐3′ and reverse, 5′‐TGTGTTTACGGTAGTGCCTG‐3′; GAPDH, forward, 5′‐CCATGGGGAAGGTGAAGGTC‐3′ and reverse, 5′‐AGTGATGGCATGGACTGTGG‐3′. A comparative Ct approach was employed to estimate relative gene expression using GAPDH as an internal reference.

### 2.5 Western blot

Total protein was extracted adopting lysis buffer (Shanghai Yubo Biotechnology Co., Ltd.) and was quantified applying BCA Protein Assay Kit (Beijing Nuobolaide Technology Co., Ltd.). Proteins (30 μg/lane) were separated by 12% sodium dodecyl sulfate‐polyacrylamide gel electrophoresis (SDS‐PAGE), and then transferred to polyvinylidene fluoride (PVDF) membranes. After being blocked with skimmed milk, the membranes were probed with primary antibodies against GPR30 (ab39742, Abcam), p‐p65 (#3031, Cell Signaling Technology), p65 (#8242, Cell Signaling Technology), Cox2 (ab179800, Abcam), iNOS (ab178945, Abcam), Bcl‐2 (ab32124, Abcam), Bax (ab32503, Abcam), cleaved‐PARP (ab32064, Abcam), PARP (ab32138, Abcam), Claudin‐1 (ab211737, Abcam), Occludin‐1 (ab216327, Abcam), ZO‐1 (ab216880, Abcam), KLF4 (ab215036, Abcam), nod‐like receptor pyrin 3 (NLRP3; ab263899, Abcam), Cleaved‐caspase1 (#4199, Cell Signaling Technology), caspase1 (#3866, Cell Signaling Technology), N‐terminal Gasdermin D (GSDMD‐N; ab215203, Abcam), and GAPDH (ab9485, Abcam) at 4°C overnight, after which was the cultivation with horse radish peroxidase (HRP)‐conjugated secondary antibody (ab6721, Abcam) at room temperature for 2 h. The visualization and analysis of protein blots were operated employing enhanced chemiluminescence and Image Lab™ software 5.2.1 (Bio‐Rad Laboratories, Inc), respectively.

### 2.6 Cytokines assay

The levels of tumor necrosis factor (TNF)‐α, interleukin (IL‐6), IL‐1β, and IL‐18 were assessed employing enzyme‐linked immunosorbent assay (ELISA). The detection process was performed in line with the guidelines of the manufacturer (Wuhan Huamei Bioengineering Institute).

### 2.7 Terminal deoxynucleotidyl transferase dUTP nick end labeling assay

Caco‐2 cells were seeded on six‐well plates and exposed to experimental procedures. After washing, the cells were exposed to 4% paraformaldehyde for fixation as well as 0.2% Triton X‐100 for permeation. Then, a terminal deoxynucleotidyl transferase dUTP nick end labeling (TUNEL) assay Kit (Roche Diagnostics) was applied for measuring the apoptotic cells strictly in line with the manufacturer's instructions. Finally, DAPI was utilized for the staining of cell nuclei and a confocal laser microscope (Leica, TCS SP5) was adopted for the capture of images.

### 2.8 Immunofluorescence

Caco‐2 cells were seeded on six‐well plates and exposed to experimental procedures. After washing, the cells were exposed to 4% paraformaldehyde for fixation as well as 0.2% Triton X‐100 for permeation. After that, cells were blocked with 5% bovine serum albumin solution for 1 h, and then cells were incubated with primary antibody against GSDMD‐N (DF12275, Affinity Biosciences) overnight at 4°C, following which was the probe with secondary anti‐rabbit IgG‐Alexa Fluor 488 antibody (#4412, Cell Signaling Technology) for 1 h at 37°C in the dark. Finally, DAPI solution was added to the cells away from light. Immunofluorescence photographs were acquired employing a confocal laser microscope (Leica, TCS SP5).

### 2.9 Transepithelial electrical resistance assay

The cells in the logarithmic growth phase were inoculated into a 0.4‐μm 24‐well transwell chamber (Costar Incorporated) at a density of 1 × 10^6^ cells/mL and exposed to experimental procedures. With the application of Millicell‐ERS device (Beijing Unique Biotechnology Co., Ltd.), the resistance was estimated. The calculation of transepithelial electrical resistance (TEER) was decided in light of the formula: TEER = (measured value – blank value) × single cell layer surface area (cm^2^).^17^

### 2.10 Dual‐luciferase reporter assay

Through referring to the human TFDB website (http://bioinfo.life.hust.edu.cn/HumanTFDB/#!/), a potential binding relationship between GPR30 promoter and KLF4 was predicted. To verify it, dual‐luciferase reporter assay was conducted. Wild‐type (WT) and mutant (MUT) GPR30 promoter dual‐luciferase reporter vectors were constructed at the KLF4 binding site of the 3′‐UTR region of GPR30 promoter. The well‐grown Caco‐2 cells were inoculated into 96‐well plates and cultured overnight. The transfection of above vectors and oe‐KLF4 or oe‐NC into Caco‐2 cells was operated by adopting Lipofectamine 3000 (Wuhan Kehaojia Biotechnology Co., Ltd.). Forty‐eight hours post‐transfection, the Dual‐Luciferase Reporter Assay system (Promega Corporation) was applied to evaluate luciferase activity. Renilla activity was used as an internal reference.

### 2.11 Chromatin immunoprecipitation assay

Caco‐2 cells were cross‐linked by applying 1% formaldehyde. Glycine was then added to terminate the cross‐linking. The collection and resuspension of cells were implemented in SDS lysis buffer. The cell lysate was sonicated to DNA fragment on ice. Subsequently, 2% supernatant served as input, and the other supernatant was immunoprecipitated by incubation with anti‐KLF4 or IgG antibodies at 4°C overnight. Eventually, the DNA was obtained through phenol/chloroform extraction and ethanol precipitation, and real‐time quantitative PCR was carried out as aforementioned.

### 2.12 Statistical analysis

All data that were displayed in the form of mean ± standard deviation (SD) got analyzed employing GraphPad Prism 8.0 (GraphPad Software). One‐way ANOVA followed by the Tukey's multiple comparison test was adopted for the comparisons among groups, while comparisons between the two groups were conducted utilizing unpaired Student's *t* test. The level of statistical significance was set at *p* < .05.

## 3 RESULTS

### 3.1 GPR30 is downregulated in Caco‐2 cells exposed to OGD/R

**Figure 1:**
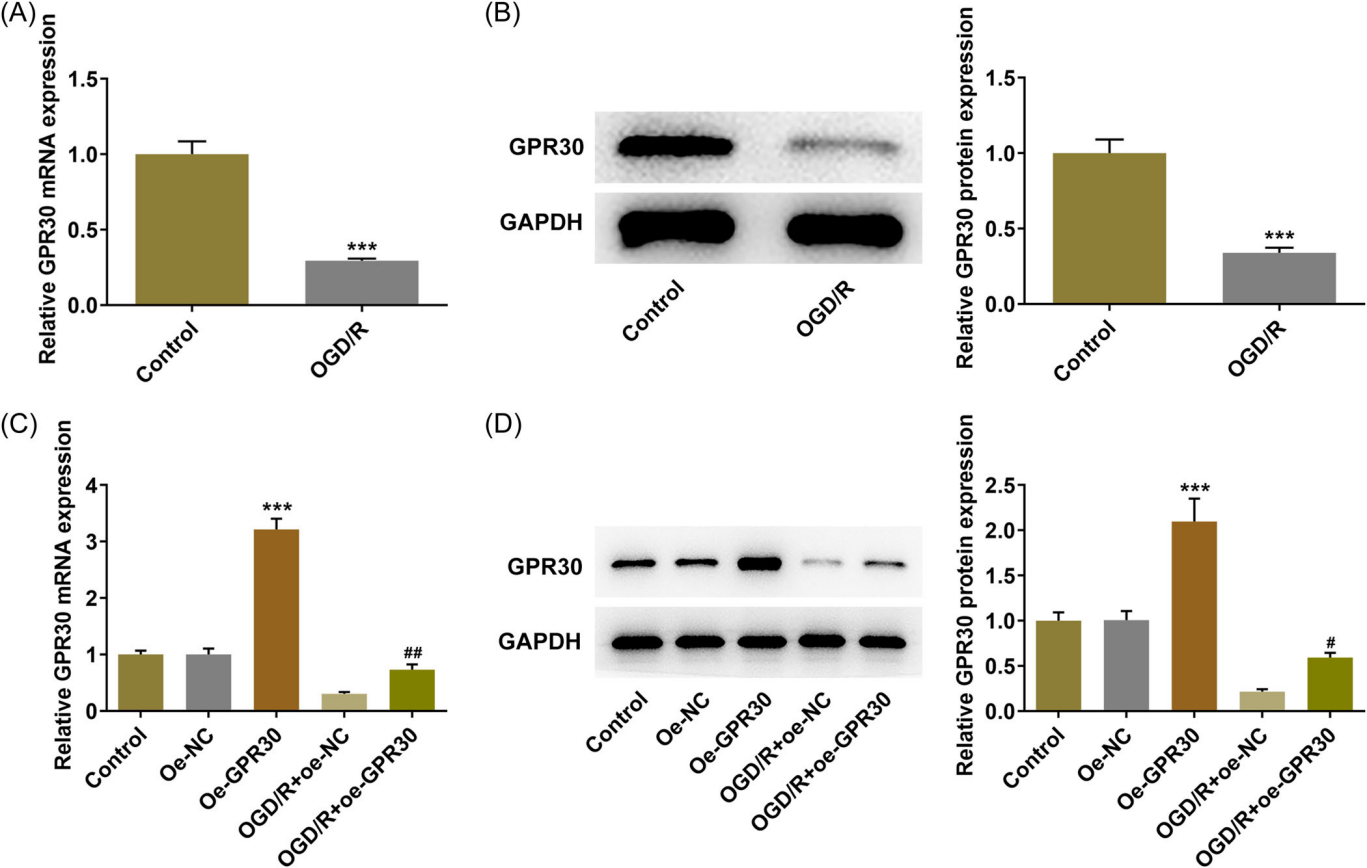
GPR30 is downregulated in Caco‐2 cells exposed to OGD/R. An OGD/R cell model was established to mimic the pathological process of I/R, and the (A) mRNA level and (B) protein expression of GPR30 were detected using RT‐qPCR and western blot, respectively. ***p < .001. Caco‐2 cells were transfected with oe‐NC or oe‐GPR30, and the un‐transfected cells and the transfected cells were exposed to OGD/R. Subsequently, the (C) mRNA level and (D) protein expression of GPR30 were detected using RT‐qPCR and western blot, respectively. OGD/R, oxygen‐glucose deprivation/reoxygenation; RT‐qPCR, real‐time quantitative PCR; ***p < .001 versus oe‐NC; #*p* < .05, ##*p* < .01 versus OGD/R+oe‐NC.

To figure out the role of GPR30 playing in intestinal I/R injury, an OGD/R cell model was established to mimic the pathological process of I/R, and GPR30 level was firstly estimated. As Figure 1A,B depicted, in comparison with the control group, both the protein expression and the mRNA level of GPR30 in the OGD/R group were downregulated. Thus, to investigate the regulatory role of GPR30 playing in intestinal I/R, cell transfection was conducted to overexpress GPR30 (Figure 1C,D).

### 3.2 GPR30 blocks OGD/R‐induced inflammation and apoptosis in Caco‐2 cells

**Figure 2:**
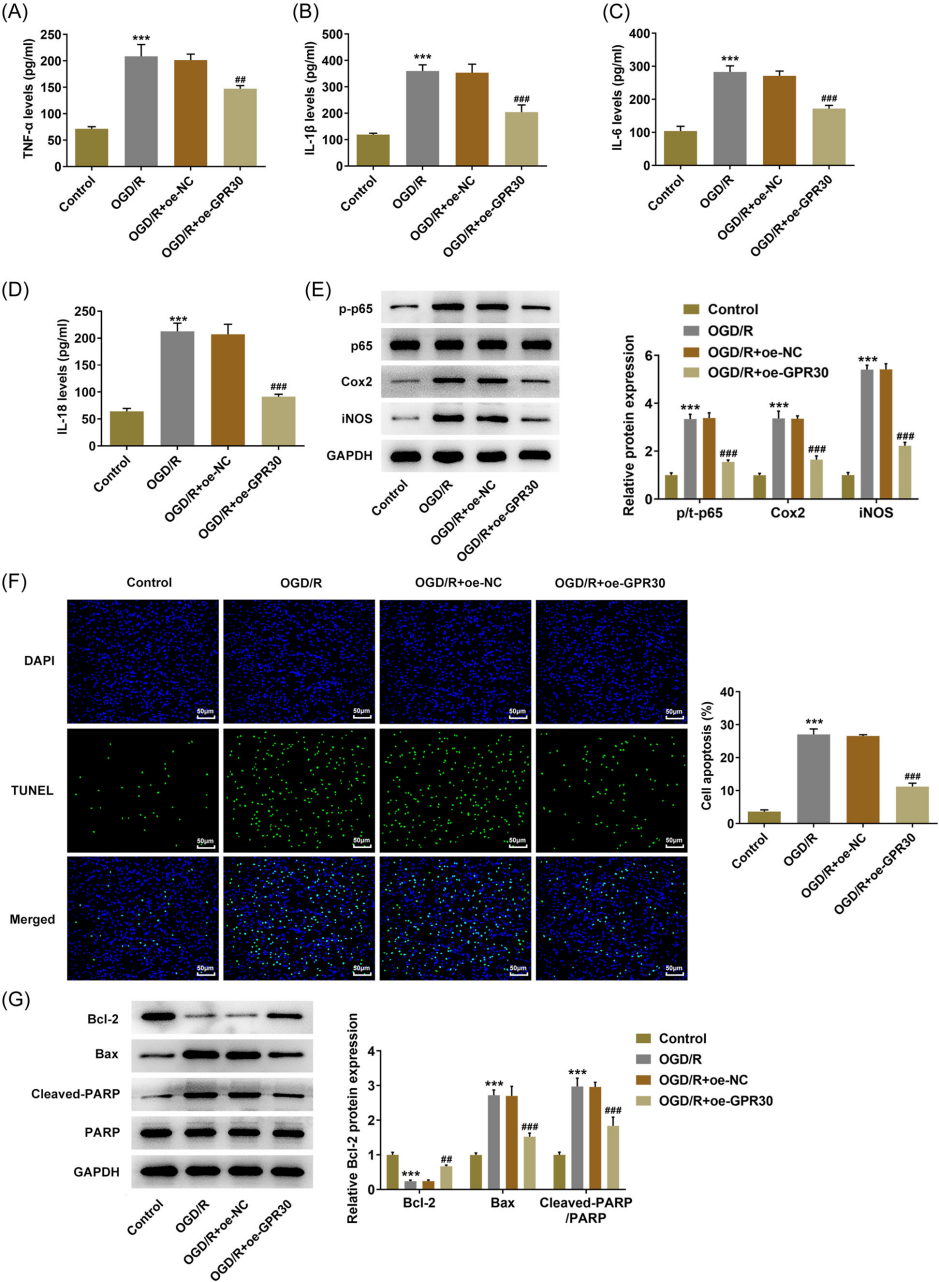
GPR30 blocks OGD/R‐induced inflammation and apoptosis in Caco‐2 cells. Untransfected and transfected Caco‐2 cells were exposed to OGD/R, and the production of pro‐inflammatory cytokines, including (A) TNF‐α, (B) IL‐6, (C) IL‐1β, and (D) IL‐18 was assessed by ELISA. (E) The protein expression of p‐p65, p65, Cox‐2, and iNOS was detected using western blot. (F) TUNEL assay was applied to evaluate cell apoptosis. (G) The protein expression of Bcl‐2, Bax, cleaved‐PARP, and PARP was detected using western blot. ELISA, enzyme‐linked immunosorbent assay; OGD/R, oxygen‐glucose deprivation/reoxygenation; TUNEL, terminal deoxynucleotidyl transferase dUTP nick end labeling. ***p < .001 versus control; ##*p* < .01, ###*p* < .001 versus OGD/R+oe‐NC.

Subsequently, to investigate the regulatory role of GPR30 playing in intestinal I/R, untransfected and transfected Caco‐2 cells were exposed to OGD/R, and the release of pro‐inflammatory cytokines was evaluated. As Figure 2A–D demonstrated, OGD/R caused a great elevation of the production of TNF‐α, IL‐6, IL‐1β, and IL‐18, while the excessive production of the cytokines in OGD/R‐exposed Caco‐2 cells was partially reduced following GPR30 overexpression, suggesting that GPR30 repressed OGD/R‐stimulated inflammatory response in Caco‐2 cells, which was further evidenced by the inhibitory effect of GPR30 overexpression on the elevated protein expression of p‐p65, Cox‐2 and iNOS in OGD/R‐exposed Caco‐2 cells (Figure 2E). Increases in iNOS transcribed by NF‐κB have been known to initiate cell apoptosis.^18^ As expected, a huge increase of TUNEL‐positive cells was observed in Caco‐2 cells exposed to OGD/R, which was partially overturned following GPR30 overexpression, suggesting that GPR30 overexpression protected Caco‐2 cells against OGD/R‐induced apoptosis (Figure 2F). Furthermore, the content of antiapoptotic protein Bcl‐2 was observed to be decreased in the OGD/R group, but was upregulated in the OGD/R+oe‐GPR30 group. Conversely, the contents of proapoptotic proteins Bax and cleaved‐PARP presented an opposite trend to Bcl‐2 (Figure 2G).

### 3.3 GPR30 restores intestinal integrity in OGD/R‐exposed Caco‐2 cells

**Figure 3:**
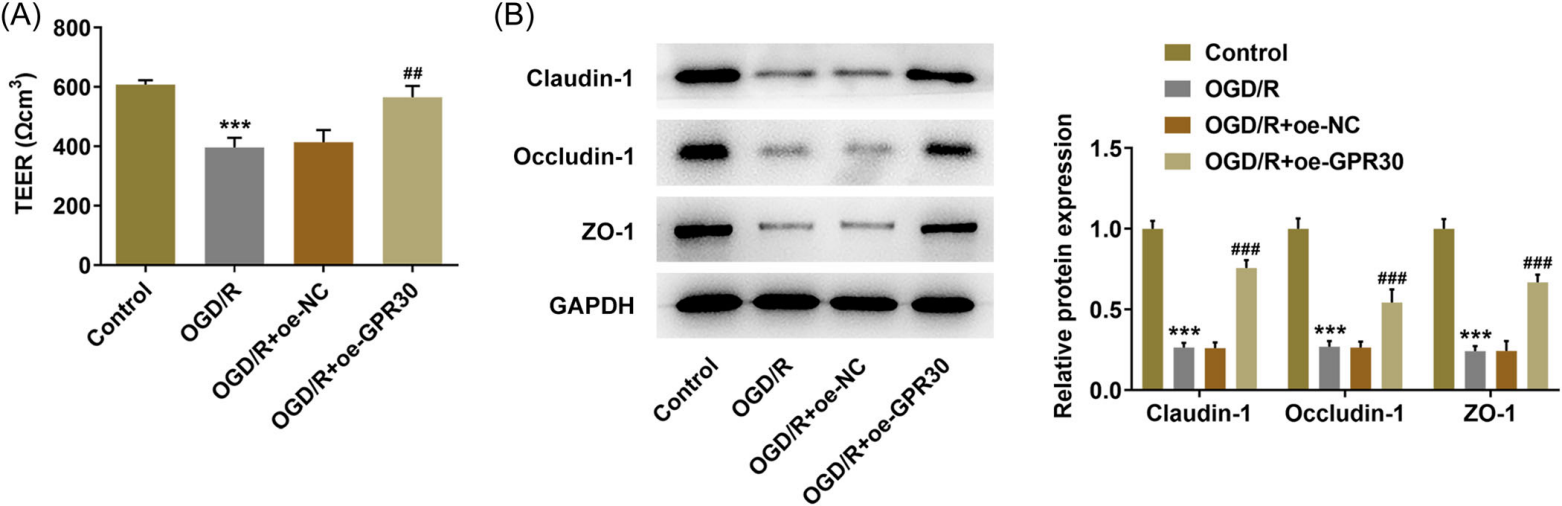
GPR30 restores intestinal integrity in OGD/R‐exposed Caco‐2 cells. (A) Untransfected and transfected Caco‐2 cells were exposed to OGD/R, and the integrity of the Caco‐2 cell monolayer was evaluated via detecting TEER. (B) the protein expression levels of claudin‐1, occludin‐1 and ZO‐1 were measured using western blot. OGD/R, oxygen‐glucose deprivation/reoxygenation; TEER, transepithelial electrical resistance. ***p < .001 versus control; ##*p* < .01, ###*p* < .001 versus OGD/R+oe‐NC.

Then, the regulatory role of GRP30 on intestinal integrity was explored. The integrity of the Caco‐2 cell monolayer was evaluated via detecting TEER. After exposure to OGD/R, an obvious drop in TEER was observed in Caco‐2 cells, indicating that OGD/R increased the permeability of the cell membrane. Meanwhile, this elevated permeability exposed to OGD/R was reduced by GPR30 overexpression, evidenced by the elevated TEER value in the OGD/R+oe‐GPR30 group (Figure 3A). To verify this finding, we further examined the contents of claudin‐1, occludin‐1, and ZO‐1. Results obtained from western blot exhibited that the contents of claudin‐3, claudin‐2 and ZO‐1 were greatly descended in OGD/R‐exposed Caco‐2 cells, which were partially restored by GPR30 overexpression (Figure 3B).

### 3.4 KLF4 binds to GPR30 promoter and transcriptionally regulates GPR30 expression

**Figure 4:**
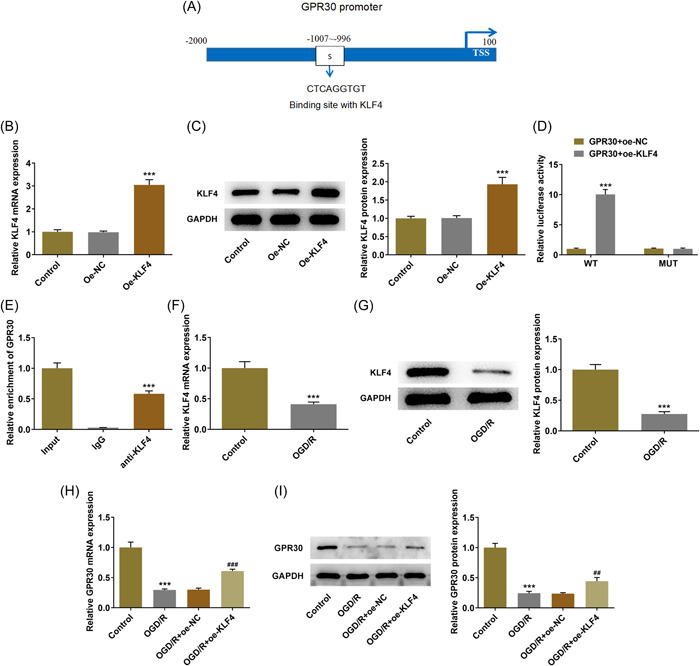
KLF4 binds to GPR30 promoter and transcriptionally regulates GPR30 expression. (A) The binding site between transcription factor KLF4 and GPR30 promoter region was predicted by human TFDB website (http://bioinfo.life.hust.edu.cn/HumanTFDB/#!/). Caco‐2 cells were transfected with oe‐NC or oe‐KLF4, and the (B) mRNA level and (C) protein expression of KLF4 was detected using RT‐qPCR and western blot, respectively. ***p < .001 versus oe‐NC. (D) Caco‐2 cells were cotransfected with GPR30‐WT/GPR30‐MUT and oe‐NC or oe‐KLF4 using Lipofectamine 3000, and the luciferase activity was examined using dual‐luciferase reporter assay kit. ***p < .001 versus GPR30+oe‐NC. (E) Chromatin immunoprecipitation assay was performed and the precipitated DNA was detected by qRT‐PCR. ***p < .001 versus IgG. (F, G) The mRNA level and protein expression of KLF4 in the control and OGD/R groups were detected using RT‐qPCR and western blot, respectively. (H, I) Caco‐2 cells were transfected with oe‐NC or oe‐KLF4, and then the un‐transfected cells and transfected cells were exposed to GPR30. The mRNA level and protein expression of GPR30 in the control and OGD/R groups were detected using RT‐qPCR and western blot, respectively. OGD/R, oxygen‐glucose deprivation/reoxygenation; RT‐qPCR, real‐time quantitative PCR. ***p < .001 versus control; ##*p* < .01, ###*p* < .001 versus OGD/R+oe‐NC.

To find out the regulator mechanism of GRP30, it is predicted from human TFDB website (http://bioinfo.life.hust.edu.cn/HumanTFDB/#!/) that transcription factor KLF4 is able to bind to the GPR30 promoter region (Figure 4A), indicating that KLF4 can potentially interact with GPR30. Then, to verify this interaction, we constructed a KLF4‐overexpression plasmid (oe‐KLF4) which could elevate both protein expression and mRNA level of KLF4 (Figure 4B,C). Subsequently, the direct binding relationship between KLF4 and GPR30 was verified by luciferase report assay and chromatin immunoprecipitation assay (Figure 4D,E). Thereafter, the expression level KLF4 was found to be downregulated in Caco‐2 cells exposed to OGD/R (Figure 4F,G). Additionally, KLF4 overexpression could elevate the downregulated expression level of GPR30 in OGD/R‐exposed Caco‐2 cells (Figure 4H,I), revealing a positive regulation of KLF4 on GPR30.

### 3.5 KLF4 knockdown partially restrains the protective role of GPR30 against OGD/R‐induced inflammation, apoptosis, and intestinal integrity disruption in Caco‐2 cells

**Figure 5:**
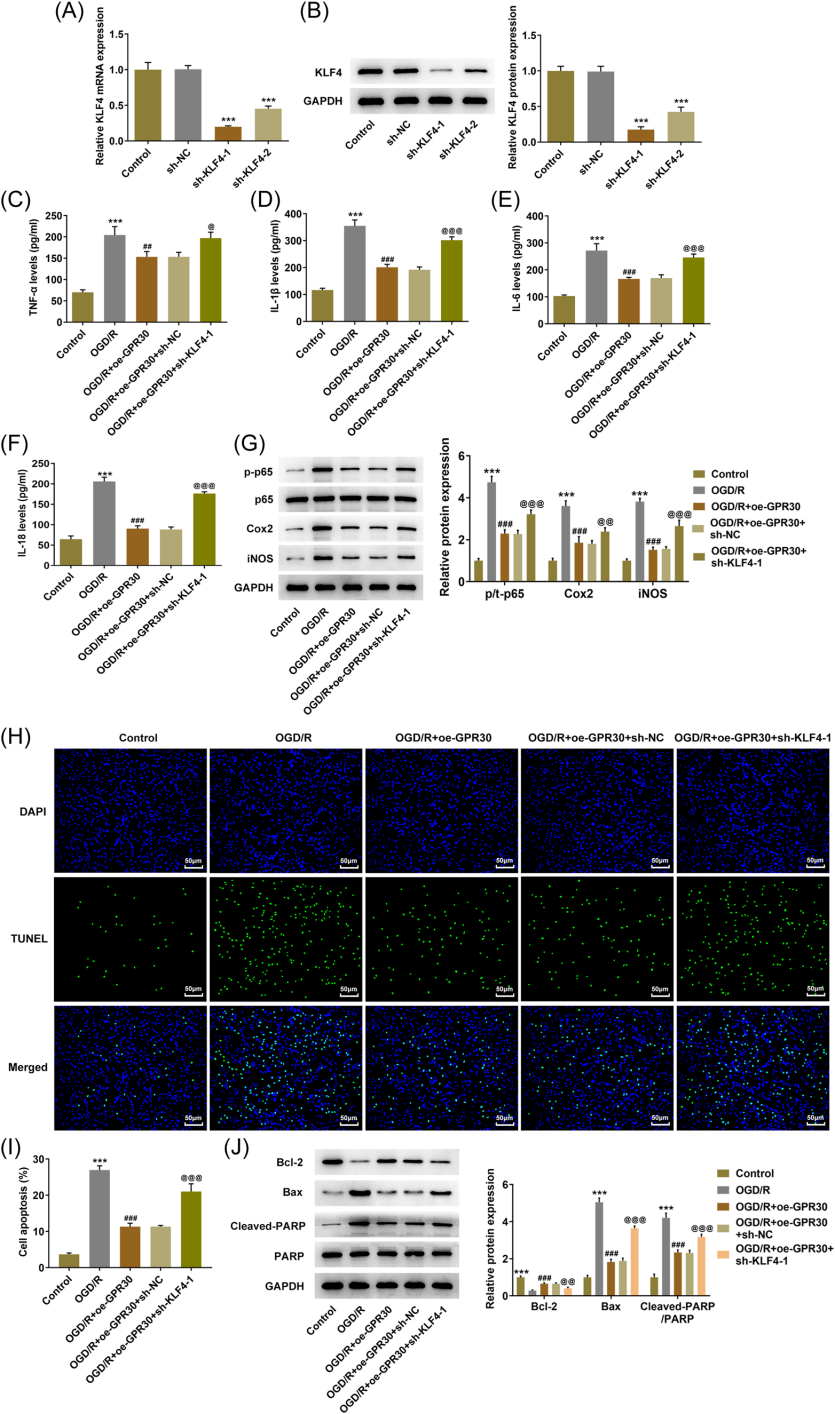
KLF4 knockdown partially restrains the protective role of GPR30 against OGD/R‐induced inflammation and apoptosis in Caco‐2 cells. Caco‐2 cells were transfected with sh‐KLF4‐1 or sh‐KLF4‐2, and the (A) mRNA level and (B) protein expression of KLF4 were detected using RT‐qPCR and western blot, respectively. ***p < .001 versus sh‐NC. Then, Caco‐2 cells were transfected with oe‐GPR30 alone or cotransfected with oe‐GPR30 and sh‐NC/sh‐KLF4‐1, following OGD/R exposure. The production of pro‐inflammatory cytokines, including (C) TNF‐α, (D) IL‐6, (E) IL‐1β, and (F) IL‐18, was assessed by ELISA. (G) The protein expression of p‐p65, p65, Cox‐2, and iNOS was detected using western blot. (H, I) TUNEL assay was applied to evaluate cell apoptosis. (J) The protein expression of Bcl‐2, Bax, cleaved‐PARP, and PARP was detected using western blot. OGD/R, oxygen‐glucose deprivation/reoxygenation; RT‐qPCR, real‐time quantitative PCR. ***p < .001 versus control; ##*p* < .01, ###*p* < .001 versus OGD/R; ^@^ *p* < .05, ^@@^ *p* < .01, ^@@@^ *p* < .001 versus OGD/R+oe‐GPR30+sh‐NC.

**Figure 6:**
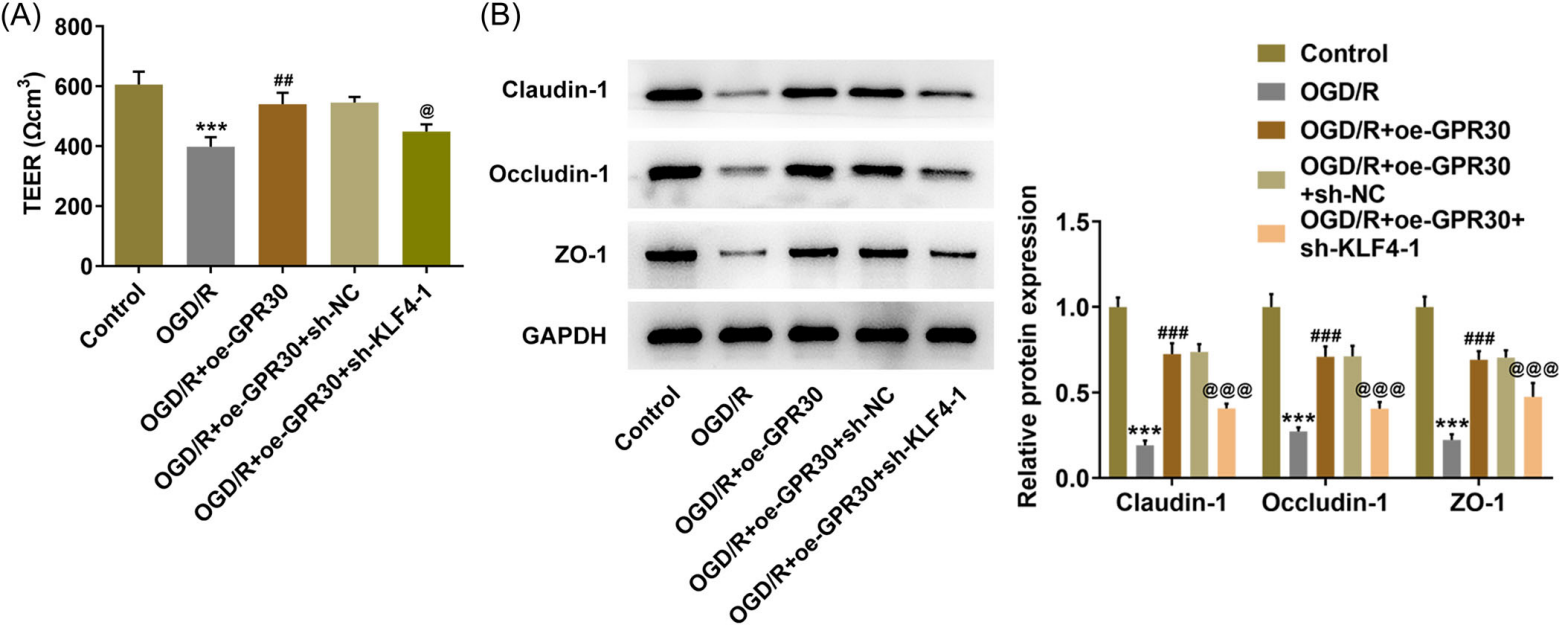
KLF4 knockdown partially restrains the protective role of GPR30 against intestinal integrity disruption in Caco‐2 cells. (A) Untransfected and transfected Caco‐2 cells were exposed to OGD/R, and the integrity of the Caco‐2 cell monolayer was evaluated via detecting TEER. (B) The protein expression levels of claudin‐1, occludin‐1, and ZO‐1 were measured using western blot. OGD/R, oxygen‐glucose deprivation/reoxygenation; TEER, transepithelial electrical resistance. ***p < .001 versus control; ##*p* < .01, ###*p* < .001 versus OGD/R; ^@^ *p* < .05, ^@@@^ *p* < .001 versus OGD/R+oe‐GPR30+sh‐NC.

Next, we further explored the regulation between GPR30 and KLF4 based on cellular biological activities. First, to achieve KLF4 knockdown, Caco‐2 cells were transfected with sh‐KLF4‐1 or sh‐KLF4‐2, and the expression level of KLF4 in sh‐KLF4‐1 group was lower than that in sh‐KLF4‐2; thus sh‐KLF4‐1 was used for the follow‐up experiments attributed to its higher transfection efficacy (Figure 5A,B). Then, Caco‐2 cells were transfected with oe‐GPR30 alone or cotransfected with oe‐GPR30 and sh‐NC/sh‐KLF4‐1. ELISA assays revealed that the reduced production of TNF‐α, IL‐6, IL‐1β and IL‐18 following GPR30 overexpression in OGD/R‐exposed Caco‐2 cells was partially abolished by the simultaneous transfection with oe‐GPR30 and sh‐KLF4‐1 (Figure 5C–F). Combined with the similar trends of p‐p65, cox‐2, and iNOS expressions following different treatments to the cytokines above (Figure 5G), it was demonstrated that KLF4 knockdown partially weakened the inhibitory effects of GPR30 on OGD/R‐induced inflammation on Caco‐2 cells. In addition, the following TUNEL assay and western blot analysis also disclosed that KLF4 knockdown weakened the inhibitory effect of GPR30 overexpression on cell apoptosis, evidenced by enhanced TUNEL‐positive cells, reduced Bcl‐2 content as well as elevated contents of Bax and cleaved‐PARP in OGD/R+oe‐GPR30+sh‐KLF4‐1 group, compared to OGD/R+oe‐GPR30+sh‐NC group (Figure 5H–J). Furthermore, the simultaneous application of oe‐GPR30 and sh‐KLF4 in OGD/R‐exposed Caco‐2 cells markedly reduced the TEER value and the protein expression claudin‐1, occludin‐1 and ZO‐1, compared to the application of oe‐GPR30 alone (Figure 6A,B), suggesting that the restored intestinal integrity following GPR30 overexpression was weakened by KLF4 knockdown in Caco‐2 cells exposed to OGD/R.

### 3.6 KLF4/GPR30 regulates NLRP3 inflammasome and pyroptosis in OGD/R‐exposed Caco‐2 cells

**Figure 7:**
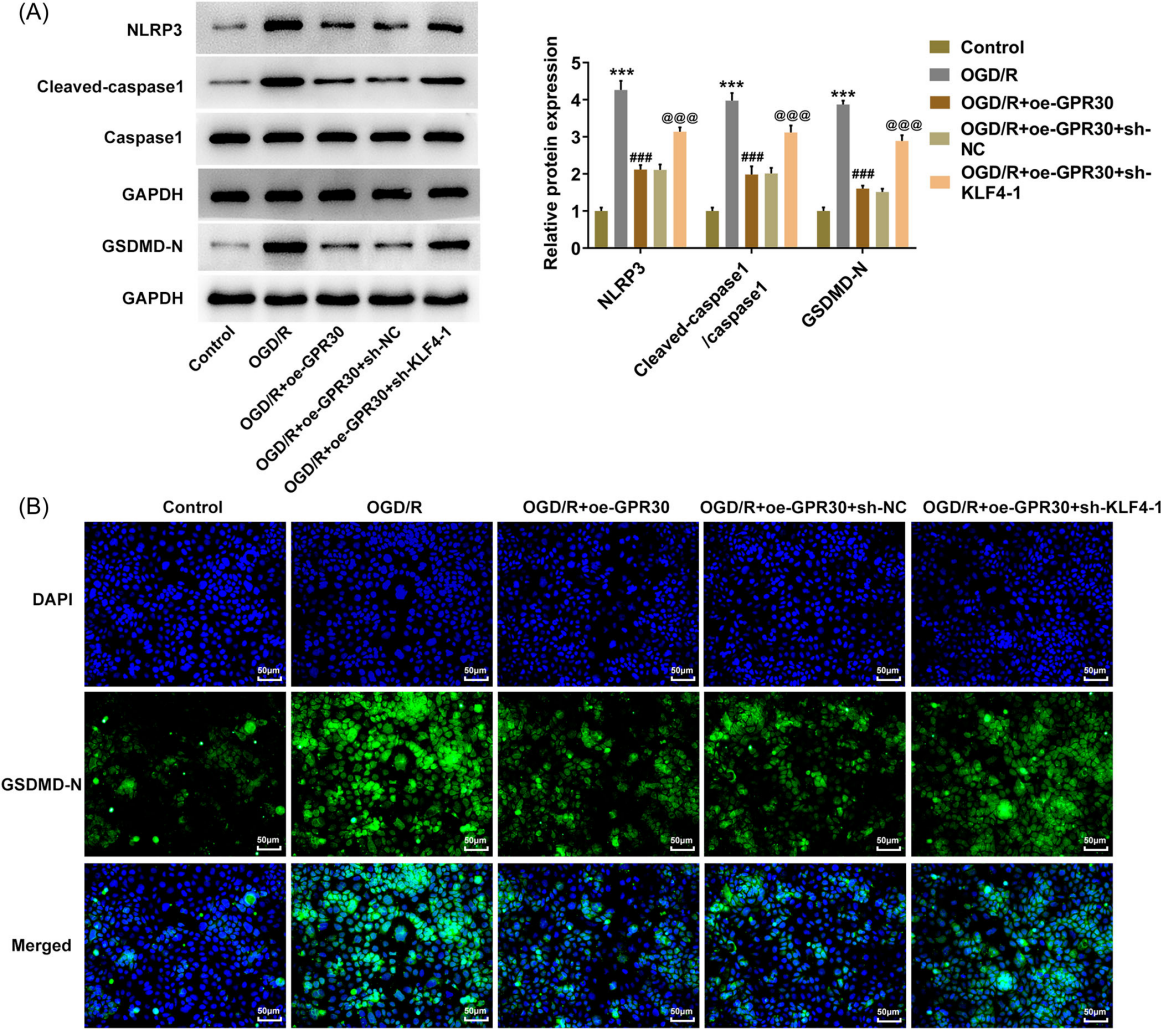
KLF4/GPR30 regulates NLRP3 inflammasome and pyroptosis in OGD/R‐exposed Caco‐2 cells. (A) Untransfected and transfected Caco‐2 cells were exposed to OGD/R, and protein expression of NLRP3, cleaved‐caspase1, caspase1, and GSDMD‐N was measured using western blot. (B) Immunofluorescence was conducted to examine GSDMD‐N expression. OGD/R, oxygen‐glucose deprivation/reoxygenation. ***p < .001 versus control; ###*p* < .001 versus OGD/R; ^@@@^ *p* < .001 versus OGD/R+oe‐GPR30+sh‐NC.

Last but not least, we explored the potential mechanism underlying the regulatory role of KLF4/GPR30 in intestinal I/R injury. As Figure 7A displayed, the contents of NLRP3 and cleaved‐caspase1 were markedly reduced in Caco‐2 cells exposed to OGD/R, which was overturned following GPR30 overexpression, revealing that GPR30 overexpression inhibited OGD/R‐activated NLRP3 inflammasome in OGD/R‐exposed Caco‐2 cells. Whereas, additional treatment of KLF4 knockdown partially weakened this function exerted by GPR30. GSDMD‐N is the executive protein of cell pyroptosis. The increased GSDMD‐N protein expression and elevated GSDMD‐N‐positive cells following OGD/R exposure revealed an activation of pyroptosis in Caco‐2 cells induced by OGD/R. However, OGD/R‐caused pyroptosis in Caco‐2 cells was repressed after overexpressing GPR30, which was then partially hindered by KLF4 knockdown (Figure 7A,B).

## 4 DISCUSSION

Intestinal I/R injury has long been a problem for clinical surgeons, and it not only induces local intestinal injury but also leads to remote organs injury, contributing to systemic complications, accompanied with high mortality.^19^, ^20^ In the present study, we first reported the following observations: (1) GPR30 protects against OGD/R‐induced inflammatory response, apoptosis as well as intestinal integrity disruption. (2) Transcription factor KLF4 directly binds to GPR30 promoter and positively regulates GPR30 overexpression. (3) KLF4/GPR30 axis regulates NLRP3‐mediated pyroptosis during the intestinal I/R injury.

Intestinal barrier breakdown has been implicated as an important turning point in intestinal I/R injury.^21^ Intestinal barrier breakdown is featured as aberrantly increased intestinal permeability. Tight junctions, including occludin, claudin, and ZO‐1, are important components of the intestinal mucosal barrier against the penetration of endotoxins and bacteria through the epithelium. Of note, the deficiency of tight junctions has been recognized as a critical factor accounting for the increased intestinal permeability, and restoration of tight junctions may alleviate this condition.^22^, ^23^ It is reported by Jiang et al. that hydrogen‐rich saline protects rats against intestinal I/R injury by maintaining the intestinal integrity and reducing intestinal epithelial tight junction barrier.^24^ Tian et al. found that curcumin protected the intestine from I/R injury through promoting the recovery of intestinal permeability and the enhancing ZO‐1 protein expression.^25^ As expected, we also discovered an elevated intestinal permeability, evidenced by the reduced TEER value and the reduced protein expression of claudin‐1, occludin‐1, and ZO‐1 in Caco‐2 cells exposed to OGD/R. GPR30 overexpression greatly restored TEER value and tight junction expression in OGD/R‐exposed Caco‐2 cells, which was partially reversed by KLF4 knockdown, demonstrating that KLF4/GPR30 axis might protect against intestinal I/R injury partly through maintaining intestinal permeability.

**Figure 8:**
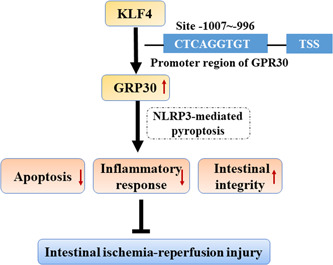
Schematic diagram of KLF4‐upregulated GPR30 might exert a protective role against intestine I/R injury by inhibiting inflammation and apoptosis, and maintaining intestinal integrity, thereby leading to the reduction of the NLRP3‐mediated pyroptosis. I/R, ischemia/reperfusion.

Pyroptosis, which is a newly discovered programmed cell death pattern, distinguishes from apoptosis and autophagy. It is featured as a caspase‐1/4/5/11‐dependent programmed cell death pathway, and the release of a large number of pro‐inflammatory factors, ultimately leading to pore formation in the cell membrane, cytoplasmic leakage, and cell rupture.^26^, ^27^ Recent evidence suggests that pyroptosis occurs following I/R injury,^22^ consistent with the findings in our study as the expression level of GSDMD‐N, the executive protein of pyroptosis, is elevated following OGD/R exposure. Meanwhile, dexmedetomidine can play a protective role by inhibiting I/R‐induced apoptosis and pyroptosis in intestinal epithelial cells, suggesting that inhibition of pyroptosis may be an alternative therapeutic strategy to alleviate I/R injury.^22^ Notably, NLRP3‐mediated pyroptosis has also been implicated to regulate I/R injury.^28^ Activation of NLRP3 inflammasome converts pro‐caspase‐1 to cleaved caspase‐1, which cleaves GSDMD and leads to the maturation and secretion of IL‐1β and IL‐18, thus resulting in pyroptosis.^29^, ^30^ Recent studies have testified that NLRP3‐related pyroptosis has involvement in intestinal diseases. For example, NEK7 regulates the activation of NLRP3 inflammasome and the subsequent pyroptosis through the interaction between NEK7 and NLRP3, thus influencing the advancement of inflammatory bowel disease.^31^ Metformin regulates intestinal epithelial cell pyroptosis via TXNIP/NLRP3/GSDMD pathway, thereby alleviating intestinal I/R injury,^32^ revealing the importance of NLRP3‐mediated pyroptosis in intestinal I/R injury. Coincidentally, GPR30 was demonstrated to inactivate NLRP3 inflammasome in hepatic I/R injury.^9^ Here, we also found that GPR30 greatly blocked the activation of NLRP3 inflammasome in intestinal I/R injury. Elsewhere, the activated pyroptosis in OGD/R‐exposed Caco‐2 cells was also repressed after overexpressing GPR30; however, these changes caused by GPR30 were partially overturned by KLF4 knockdown, suggesting a potential mechanism that KLF4‐upregulated GPR30 might exert its functions by inhibiting NLRP3‐mediated pyroptosis (Figure 8).

## 5 CONCLUSION

In short, our study provides the first evidence of the important role of GPR30 in intestinal I/R injury. The results showed that GPR30 prevented intestine I/R injury by inhibiting inflammation, apoptosis, and maintaining intestinal integrity. In addition, GPR30 inhibited the NLRP3‐mediated pyroptosis. Furthermore, KLF4 could directly bind to GPR30 and upregulate GPR30. These findings make a notion that KLF4/GPR30 serves as a critical axis in the intestinal I/R injury and provide a new therapeutic target for the treatment of the intestinal I/R injury.

## AUTHOR CONTRIBUTIONS

**Jie Yin**: Data curation (lead); methodology (equal); formal analysis (equal); writing—original draft (lead). **Xiaoli Xie**: Data curation (equal); methodology (equal); formal analysis (equal); writing—original draft (supporting). **Jinfeng Yao**: Data curation (equal); methodology (equal); formal analysis (supporting); software (lead). **Xiaoxu Jin**: Data curation (equal); methodology (equal); formal analysis (supporting). **Huiqing Jiang**: Data curation (equal); methodology (supporting); software (supporting). **Chenguang Ji**: Conceptualization (lead); writing—review and editing (lead).

## CONFLICT OF INTEREST STATEMENT

The authors declare no conflict of interest.

## Data Availability

All data generated or analyzed during this study are included in this published article.
